# Full-Length cDNA, Prokaryotic Expression, and Antimicrobial Activity of UuHb-F-I from* Urechis unicinctus*

**DOI:** 10.1155/2016/5683026

**Published:** 2016-06-07

**Authors:** Rongli Niu, Xiang Chen

**Affiliations:** Engineering Research Center of Molecular Medicine, Ministry of Education, Huaqiao University, Xiamen 361021, China

## Abstract

Hemoglobin, which widely exists in all vertebrates and in some invertebrates, is possibly a precursor of antimicrobial peptides (AMPs). However, AMPs in the hemoglobin of invertebrates have been rarely investigated. This study is the first to report the full-length cDNA, prokaryotic expression, and antimicrobial activity of UuHb-F-I from* Urechis unicinctus*. The full-length cDNA sequence of UuHb-F-I was 780 bp with an open-reading frame of 429 bp encoding 142 amino acids. MALDI-TOF-MS suggested that the recombinant protein of UuHb-F-I (rUuHb-F-I) yielded a molecular weight of 15,168.01 Da, and its N-terminal amino acid sequence was MGLTGAQIDAIK. rUuHb-F-I exhibited different antimicrobial activities against microorganisms. The lowest minimum inhibitory concentration against* Micrococcus luteus* was 2.78–4.63 *μ*M. Our results may help elucidate the immune defense mechanism of* U. unicinctus* and may provide insights into new AMPs in drug discovery.

## 1. Introduction

Hemoglobin (Hb), which widely exists in all vertebrates and in some invertebrates, contains endogenous biologically active proteins [1] exhibiting various properties, including hormone release and immunomodulatory, hematopoietic, coronaroconstrictory, antigonadotropic, and opioid-like activities [2]. Hb is also a possible precursor of antimicrobial peptides (AMPs) [3–10]. Thus far, 30 AMPs have been derived from peptic Hb hydrolysates, 24 peptides have been obtained from the *α* chain of Hb, and 6 peptides have been obtained from the *β* chain of Hb [10, 11]. Intact Hb*α* or Hb*β* is also a potent antibacterial protein [5]. Hence, Hb-associated AMPs have been extensively investigated. However, few Hb-associated AMPs in invertebrates have been reported [12].

*Urechis unicinctus* (Uu), a marine spoon worm, is economically important seafood mainly distributed throughout Russia, Japan, Korea, and China. Uu possesses a well-developed body cavity filled with coelomic fluid, which contains cells with Hb. In general, AMPs are found in most living organisms and considered an essential component of an organism's innate immune system [13]. Thus, AMPs may be found in the Hb or coelomic fluid of Uu. AMPs may also play an important role in its innate immune system. However, the Hb of Uu and its antimicrobial activity have yet to be described. Novel AMPs or antimicrobial substances from the blood of Uu should be identified and isolated. In this study, the Hb of Uu was analyzed and its cDNA was cloned. Recombinant expression and antimicrobial activity assay were then performed. Our research on the structure and potential function of Hb may help elucidate the immune defense mechanism of invertebrates. This study may also provide insights into new AMPs for drug discovery and disease control in* U. unicinctus* aquaculture.

## 2. Materials and Methods

### 2.1. Cloning of the cDNA of UuHb-F-I Fragment

The coelomic fluid of an adult fresh Uu (about 20.5 cm in length and 30.5 g in mass) was collected and centrifuged at 12,000 rpm for 5 min at 4°C. The precipitates were collected and RNA was extracted by using a Trizol kit in accordance with the manufacturer's protocol (Shenggong Bioengineering Co., Ltd., China). First-strand cDNA was synthesized with M-MLV reverse transcriptase, oligo dT, dNTP mix, and total RNA. Then, PCR was conducted in 20 *μ*L reaction mixture containing 1 *μ*L of first-strand cDNA, 0.5 *μ*L of each primer (primers P1 and P2; Table 1), 10 *μ*L of 2x* Taq* Master Mix (Omega Bio-Tek), and 8 *μ*L of MilliQ H_2_O. Amplifications were performed on PCR 3 Block Professional Thermocycler (Biometra) under the following conditions: initial denaturation at 94°C for 3 min, 30 cycles of denaturation at 94°C for 30 s, annealing at 48°C for 30 s, extension at 72°C for 50 s, and final extension at 72°C for 10 min. The obtained cDNA was further purified with a SanPrep PCR product purification kit (Shenggong Bioengineering Co., Ltd., China) and cloned into pUM-T vector. Positive recombinants were transformed into competent DH5*α* cells, identified through anti-Amp selection, and verified through double digestion with Sal I and BamH I (Thermo Scientific). Afterward, the positive clone was sequenced (Nanjin Jinsirui Biotechnology Ltd., Co., China).

### 2.2. Full-Length cDNA Sequence Determination

#### 2.2.1. 3′-RACE

3′-RACE was performed using 3′-Full RACE Core Set with PrimeScript RTase (TaKaRa) in accordance with manufacturer's instructions. Nested PCR was conducted in 3′-RACE outer primer and 3′-RACE-GSP1 or 3′-RACE inner primer and 3′-RACE-GSP2 (Table 1). The first round of PCR was performed using a reaction mixture containing 1 *μ*L of the first-strand cDNA, 0.5 *μ*L of each primer (10 *μ*M), 2 *μ*L of 10x Trans* Taq* HiFi buffer, 2 *μ*L of dNTPs (2.5 mM), 0.3 *μ*L of Trans* Taq* HiFi DNA Polymerase (TransGen Biotech), and 13.7 *μ*L of MilliQ H_2_O. The second round of PCR was conducted using a reaction mixture with 2 *μ*L of outer PCR purified product, 1 *μ*L of each primer (10 *μ*M), 5 *μ*L of 10x Trans* Taq* HiFi buffer, 4 *μ*L of dNTPs (2.5 mM), 0.5 *μ*L of Trans* Taq* HiFi DNA polymerase, and 36.5 *μ*L of MiliQ H_2_O. The amplifications of the first round were performed with initial denaturation at 94°C for 3 min, 30 cycles with denaturation at 94°C for 30 s, annealing at 48°C for 30 s, extension at 72°C for 50 s, and the final extension step at 72°C for 10 min. The second round was performed in the same manner as that of the first round except annealing at 56°C. The inner PCR product was ligated with pUM-T vector and further purified and transformed into DH5*α*. The detailing process was the same as above. The sequence was then determined (Nanjin Jinruisi Biotechnology Ltd., Co., China).

#### 2.2.2. 5′-RACE

5′-RACE was performed using 5′-Full RACE kit with TAP (TaKaRa) in accordance with the manufacturer's instructions. Nested PCR was conducted with 5′-RACE outer primer and 5′-RACE-GSP1 or 5′-RACE inner primer and 5′-RACE-GSP2. The PCR system in the first round contained 2 *μ*L of reverse transcriptase, 1 *μ*L of each primer, 5 *μ*L of 10x Trans* Taq* HiFi buffer, 4 *μ*L of dNTP (2.5 mM), 0.5 *μ*L of Trans* Taq* HiFi DNA polymerase, and 36.5 *μ*L of MilliQ H_2_O. The touchdown PCR profile was as follows: initial denaturation at 94°C for 3 min; 30 cycles at 94°C for 30 s, at 60°C for 30 s (decreased by 0.5°C in each cycle), and at 72°C for 1 min; 10 cycles at 94°C for 30 s, at 45°C for 30 s, and at 72°C for 1 min; final extension at 72°C for 10 min; and being terminated at 15°C. The inner PCR was performed using 1 *μ*L of the purified outer PCR product, 1 *μ*L of each primer, 5 *μ*L of 10x Trans* Taq* HiFi buffer, 4 *μ*L of dNTPs (2.5 mM), 0.5 *μ*L of Trans* Taq* HiFi DNA polymerase, and 37.5 *μ*L of MilliQ H_2_O. The touchdown PCR was performed using the following parameters: 94°C for 3 min; 30 cycles at 94°C for 30 s, at 66°C for 30 s (decreased by 0.5°C in each cycle), and at 72°C for 40 s; 10 cycles at 94°C for 30 s, at 51°C for 30 s, and at 72°C for 40 s; final extension at 72°C for 10 min; and being terminated at 15°C. After the results were verified through electrophoresis, the product was sequenced to obtain the full length of UuHb-F-I cDNA.

### 2.3. Bioinformatics Analysis

Bioinformatics was conducted to predict the new gene and the conservation, consistency, and structure of the mature peptide. The homology of nucleotide and protein sequences was blasted by using an online tool at the National Center for Biotechnology Information (http://blast.ncbi.nlm.nih.gov/Blast.cgi). The deduced amino acid sequence was analyzed by using a translate tool (http://web.expasy.org/translate/). Clustal X and DNAman were used to perform multiple alignments of amino acid sequences. The presence and location of a signal peptide were predicted by using SignalP 4.1 Server online. ProtScale (Hphob/Kyte & Doolittle), Sopma, and Phyre2 online software were adopted to analyze possible amphiphytes and structures.

### 2.4. Expression and Purification of Recombinant UuHb-F-I

#### 2.4.1. Construction of Recombinant UuHb-F-I

The CDS sequence, encoding mature peptide of UuHb-F-I, was amplified by a pair of primers (CDS-P1 and CDS-P2). The PCR product and pET-22b^+^ plasmids were double-digested with Nde I and Xho I (Thermo Scientific). Afterward, the purified product was inserted into pET-22b^+^ vector by the T4 ligation enzyme. The ligation product was transformed into competent BL21(DE3) cells and sequenced to ensure in-frame insertion. Blank pET-22b^+^ plasmids were used as a negative control.

#### 2.4.2. Expression and Determination of Recombinant Protein

BL21(DE3)/pET-22b^+^ and BL21(DE3)/pET22b-UuHb-F-I were inoculated in a TB medium with Amp (100 *μ*g/mL) at 200 rpm and 37°C until OD_600_ of 0.6–0.8 was reached. Isopropyl-*β*-d-thiogalactosidase (IPTG, 100 mM) was added to induce expression under the same conditions. The cells were harvested through centrifugation at 12,000 rpm for 1 min. Inducing conditions, including the final IPTG concentration and induction time, were optimized.

Lactose instead of IPTG was used to induce protein expression. The positive transformants of UuHb-F-I and the negative control were incubated in an FML medium composed of 15 g/L tryptone, 12 g/L yeast extract, 3 g/L NaH_2_PO_4_·2H_2_O, 7 g/L K_2_HPO_4_·3H_2_O, 2.5 g/L NaCl, 0.2% glucose, 2.1 mM lactose, 0.05% MgSO_4_·7H_2_O, and 100 g/mL Amp at 37°C with shaking at 180 rpm in accordance with the procedure involving IPTG. Lactose was added to induce expression; the cells were then harvested. The induction time obtained using lactose was compared with that recorded using IPTG. The quantities of the expressed proteins were compared through SDS-PAGE.

The recombinant protein of UuHb-F-I (rUuHb-F-I) was further confirmed through Western blot analysis. After SDS-PAGE was conducted, the proteins were transferred from the gel to a PVDF film. The film was blocked with 5% fat-free milk, inoculated with His-Tag (27E8) mouse mAb (Cell Signaling) and peroxidase-conjugated AffiniPure goat anti-mouse IgG (H+L) (Shenggong Bioengineering Co., Ltd., China), and colored with a stable peroxide solution (A) and a luminol/enhancer solution (B). Images were captured using ChemiDoc MP imaging system (Bio-Rad).

#### 2.4.3. Purity and Renaturation of Recombinant Proteins

Lactose was used to induce protein expression. The recombinant strain of pET-22b-UuHb-F-I was inoculated in an LB medium, transferred to 100 mL of FML in a 1 L flask, and cultivated for 16 h at 37°C with 180 rpm. The cultivation solution was centrifuged at 10,000 rpm for 10 min. The pellet was solubilized with cell lysates (0.5 M NaCl, 50 mM Tris-HCl, 1 mM EDTA, and 0.5% Triton X-100, pH 7.4). The solution was sonicated for 20 min with 2 s ultrasonication and 2 s intervals at 400 W power and centrifuged at 10,000 rpm and 4°C for 20 min. The pellet contained inclusion bodies, which were further washed with buffer I (0.5 M NaCl, 50 mM Tris-HCl, 2 M urea, 0.5% Triton X-100, and 1 mM EDTA, pH 7.4) and dissolved in buffer II (0.5 M NaCl, 50 mM Tris-HCl, 8 M urea, and 1 mM EDTA, pH 7.4). The supernatant was prepared for column purification. The samples from each step subjected to SDS-PAGE to determine the target protein. rUuHb-F-I was purified with Ni^+^ affinity resins under denaturation conditions.

The purified proteins were renatured through dialysis in the following: gradient urea glycerol buffer (0.5 M NaCl, 50 mM Tris-HCl, 1% glycine, 10% glycerol, 1 mM EDTA, and a gradient concentration of 4, 2, and 1 M urea in each gradient, pH 7.4; each gradient for 4 h); PBS for 4 h; and deionized water for 8 h. The sample was cold-dried and analyzed through SDS-PAGE.

### 2.5. Determination of the Molecular Weight and Amino Sequence of the Purified rUuHb-F-I

The molecular weight of the purified rUuHb-F-I was confirmed by using an ABI 5800 MALDI-TOF/TOF plus mass spectrometer (AB SCIEX) operated in a linear mode at Boyuan Bio-Tech Co. (Shanghai, China). MS and MS/MS data were integrated and analyzed in GPS Explorer V3.6 (Applied Biosystems, USA) with default parameters. The MS/MS spectra revealed that proteins were successfully obtained, as indicated by ≥95% confidence interval of their scores in MASCOT V2.3 search engine (Matrix Science Ltd., London, UK).

### 2.6. Antimicrobial Analysis

The lyophilized protein was dissolved in acetic acid (0.025%, V/V) at different concentrations: 1, 1.67, 2.78, 4.63, 7.72, 12.86, 21.4, 35.7, 59.5, and 99.2 *μ*M. The concentration of rUuHb-F-I was estimated by using a BCA protein kit (Thermo Scientific). The antimicrobial activities of eight microbial strains were measured: three Gram-positive bacteria, namely,* Staphylococcus aureus*,* Bacillus subtilis*, and* Micrococcus luteus*; four Gram-negative bacteria, namely,* Escherichia coli* (ATCC8739),* Pseudomonas aeruginosa*,* Vibrio alginolyticus*, and* Vibrio parahaemolyticus*; and one fungus, namely,* Pichia pastoris* GS115 (China General Microbiological Culture Collection Center (CGMCC, China)).* V. alginolyticus *and* P. pastoris* GS115 were cultured in TSB (17 g/L tryptone, 3 g/L soytone, 5 g/L NaCl, 2.5 g/L glucose, and 2.5 g/L K_2_HPO_4_) and YPD (2% (W/V) tryptone, 2% (W/V) d-glucose, and 1% (W/V) yeast extract) at 30°C, separately. Other bacteria were cultured in TSB at 37°C. Antibacterial activity was analyzed through a liquid phase assay, as described previously [14, 15]. The strains were initially adjusted to 10^3^ CFU/mL with LTM (1% agar in PBS); afterward, 120 *μ*L of each strain was seeded into 96-well plate, and each well contained 50 *μ*L of the protein sample. The plate was incubated for 3 h at 37°C or 30°C. Subsequently, 125 *μ*L of the medium was added to each well and cultivated for another 12 h. Then, 100 *μ*L sample from each well was spread onto plates and cultivated for 24 h. The highest growth concentration and the lowest inhibitory concentration were recorded. Minimum inhibitory concentration (MIC) was determined by using the following equation: *a* − *b*, where *a* is the highest protein concentration of bacterial growth and *b* is the lowest protein concentration that totally inhibited bacterial growth. Acetic acid (0.025%) was used as a negative control. Isopropanol (70%) was used as a positive control for* P. pastoris* GS115. Chloramphenicol solution (0.68 mg/mL) was utilized as a positive control for other bacteria. Each treatment was repeated thrice.

## 3. Results

### 3.1. cDNA Cloning and Sequence Analysis of UuHb-F-I

**Figure 1:**
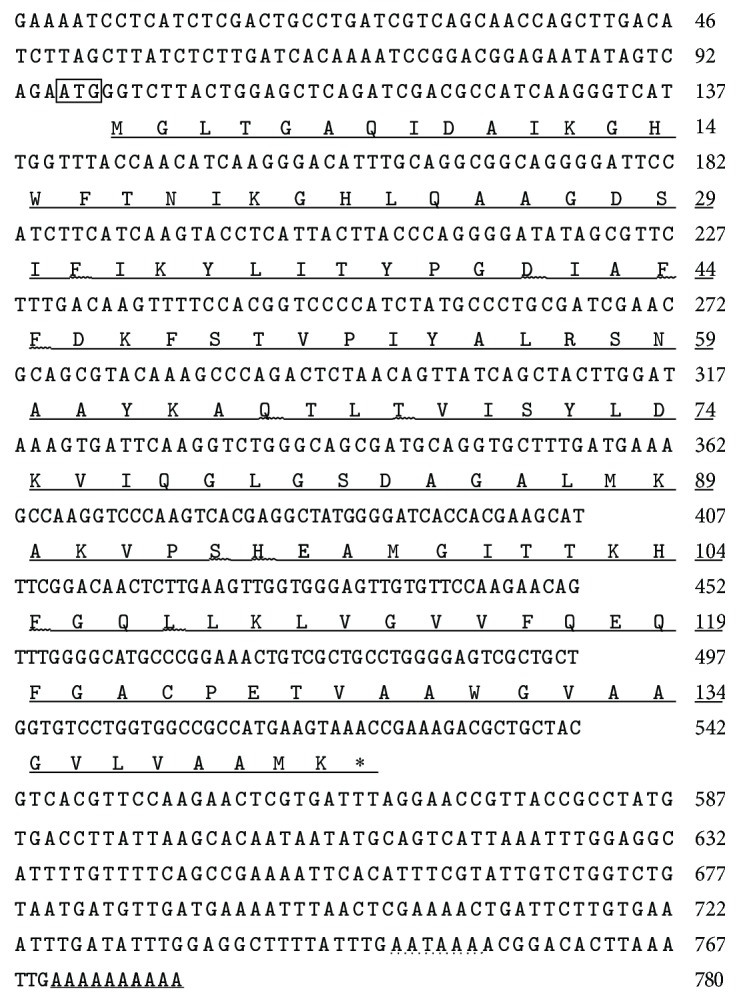
Nucleotide and deduced amino acid sequences of F-I chain of hemoglobin from* Urechis unicinctus*. The start codon (ATG) is boxed. The stop codon (TAA) is indicated by an asterisk. The polyadenylation signal motif (AATAAA) is in dotted line. The protein sequence of UuHb-F-I deduced from the nucleotide sequence is underlined. The letters underlined with a curve line are the predicted combining site of heme to protein. The poly(A) is double-underlined. Numbers on the right side of the sequence show the positions of the last nucleotide or amino acid on each line.

On the basis of* Urechis caupo *F-I complete CDS (GI:945055), we obtained the cDNA of* U. unicinctus*. The nucleotide and deduced amino acid sequences are shown in Figure 1, and the sequence data were deposited in GenBank (KJ865621).

The full-length cDNA sequence of UuHb-F-I was 780 bp. It contains 95 bp 5′-untranslated region (UTR), 256 bp 3′-UTR, and 429 bp open-reading frame (ORF) encoding 142 amino acids (AA). The poly(A) tail was found in UuHb-F-I, and a canonical polyadenylation signal sequence (AATAAA) was detected. The estimated molecular weight of mature UuHb-F-I was 15,120.67 Da, and the theoretical isoelectric point was 9.02. Moreover, numerous *α*-helices were observed in the secondary structure of mature UuHb-F-I. UuHb-F-I is amphiphilic, as analyzed by Hphob./Kyte & Doolittle in ProtScale. Signal peptide prediction revealed no signal sequences in UuHb-F-I. Using Sopma and Phyre2, we could further predict the secondary and tertiary structures of this protein (not shown in this study).

**Figure 2:**
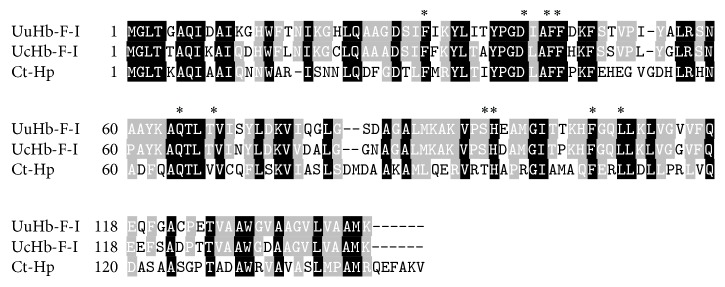
Multiple alignment of amino acid sequences of UuHb-F-I with other known globins. Amino acid residues that are conserved in the same sequences are shaded in black; similar amino acids of at least 60% are shaded in gray. Numbers on the right indicate the amino acid position of the different sequences. The heme-binding domains are marked with asterisk above the alignment. The species and the GenBank accession numbers are as follows: UuHb-F-I (*Urechis unicinctus* hemoglobin F-I), UcHb-F-I (*Urechis caupo *hemoglobin F-I, GI:122733), and Ct-Hp (*Capitella teleta* hypothetical protein, GI:443723524).

BLAST analysis revealed that the nucleotide acid and deduced amino acid sequences of UuHb-F-I matched those of UcHb-F-I, with 82%–87% and 79% similarities, respectively [16]. By contrast, the sequence similarities to other organisms were relatively low and mainly conserved in the binding site (Figure 2). UuHb-F-I displayed 43%, 36%, and 13.79% amino acid identities with* Capitella teleta* (GI:443723524),* Daphnia magna *(GI:322229317) [17], and human hemoglobin chain (GI:3114508), respectively.

### 3.2. Expression and Purification of Recombinant UuHb-F-I

The recombinant plasmids pET-22b-UuHb-F-I were transformed and expressed in* E. coli *BL21(DE3) (Tianjin, China). The results showed that the protein expression level of the inducing group was much higher than that of the noninducing group. The blank plasmid did not induce band expression; this finding suggested that BL21(DE3)/pET22b-UuHb-F-I was the actual strain that induced expression. We further optimized the IPTG inducing conditions and observed that the highest protein expression level was obtained at 1 mM IPTG and 3 h induction time. We also induced the protein expression by using lactose and found that the highest protein expression level was determined at 16 h induction time. The obtained protein expression level at 16 h was higher than that recorded at 8 or 12 h.

**Figure 3:**
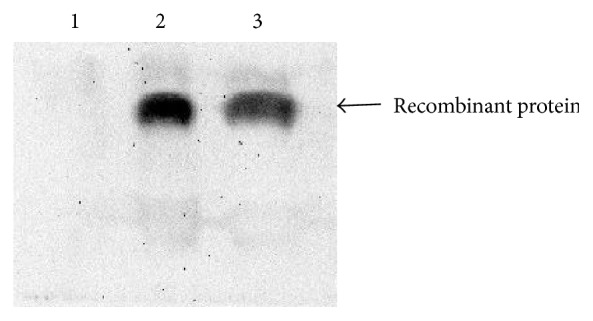
Result of Western blot for induced expression (1, negative; 2, IPTG induction; 3, lactose induction).

**Figure 4:**
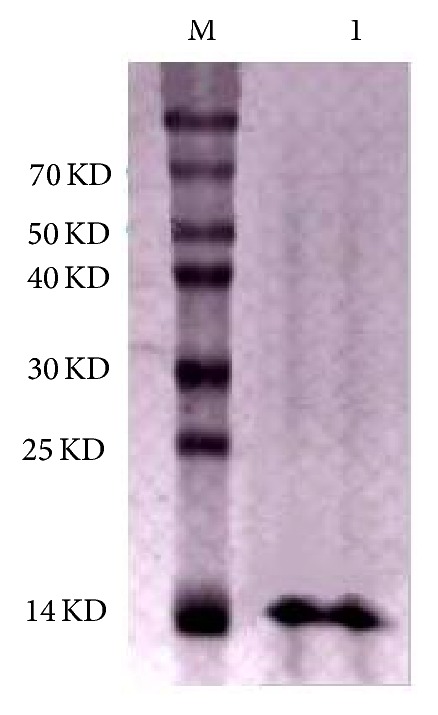
Purified recombinant protein (M: marker; 1: recombinant protein).

After induction was completed, the whole cell lysate and insoluble fraction were analyzed through SDS-PAGE. The results revealed that the recombinant UuHb-F-I was mainly expressed as insoluble proteins and accumulated in inclusion bodies. Western blot (Figure 3) demonstrated that the recombinant strain could produce recombinant proteins with His-Tag after induction was completed. This finding confirmed that the obtained protein was indeed the target protein. The target protein was purified using Ni^+^ affinity column (Figure 4), dialyzed, and cold-dried for antibacterial assay. The purified* rUuHb-F-I* was further measured by MALDI-TOF-MS/MS. The result showed that the pure peptide yielded an observed molecular mass of 15168.01 Da, and its N-terminal sequence was MGLTGAQIDAIK.

### 3.3. Antimicrobial Activities of rUuHb-F-I

The antibacterial activities of rUuHb-F-I are described in Table 2. rUuHb-F-I exhibited inhibitory activity against G^+^ and G^−^. Among the obtained MICs, the MIC against* M. luteus* was the smallest, with 2.78–4.63 *μ*M. The MIC against* S. aureus* was 7.72–12.86 *μ*M. The MIC of rUuHb-F-I against G^−^, such as* E. coli* and* P. aeruginosa*, was 35.7–59.5 *μ*M, which was higher than that of G^+^. This protein also elicited an inhibitory effect on* V. parahaemolyticus*, with MIC of 21.4–35.7 *μ*M. By contrast, this protein did not affect* V. alginolyticus* and* P. pastoris* GS115.

## 4. Discussions

This study is the first to report the full-length cDNA, prokaryotic expression, and antimicrobial activity of UuHb-F-I from* U. unicinctus*.

Sequence analysis revealed that the mature peptide of UuHb-F-I is a globin belonging to the heme protein family. UuHb-F-I contains many *α*-helices (70.42%) and heme-binding sites. These properties are similar to those of Hb in other animals [14, 16]. The nucleotide acid and deduced amino acid sequences of UuHb-F-I exhibited 82%–87% and 79% similarities to those of UcHb-F-I, respectively. The combination sites of heme with UuHb-F-I are 31 (F), 41 (D), 44 (F), 45 (F), 65 (Q), 68 (T), 94 (S), 95 (H), 105 (F), and 108 (L), which are consistent with those of UcHb-F-I. UcHb-F-I contains 137 (L) sites, but UuHb-F-I does not consist of these sites. Therefore, Uu and Uc were derived from the same descendent, and their Hb-F-I was the same.

The mechanism of AMPs shows that positive charges and amphiphilic *α*-helices are common molecular structures which accounted for their antimicrobial activity [18, 19]. Zhu et al. [15] reported that *α*-helices in peptides and charges are responsible for antimicrobial activities; changes in amphiphilicity can affect antimicrobial properties. Giangaspero et al. [20] suggested that antimicrobial activities may be decreased by reducing the positive charges or the number of *α*-helices. Our results showed that UuHb-F-I contains many *α*-helices (70.42%). Therefore, UuHb-F-I could exhibit antimicrobial activity. Uu with a unique Hb can live in active pathogenic zones, such as muds and burrows in sand, because of this property and thus protect themselves from other microbial invasions.

As a strong inducer, IPTG can induce high protein productivity at low doses. In this study, the expression level increased as IPTG concentration increased within a certain range, and the maximum product was obtained at 1 mM IPTG after 3 h of induction. However, IPTG might be replaced with lactose because of its high costs and toxicity. Lactose can produce the same or greater expression level than that of IPTG [21–23]. Our result indicated that lactose could induce the expression of relatively pure proteins and thus simplify purification. rUuHb-F-I was purified and further quantified through MALDI-TOF-MS/MS. The result revealed that the pure peptide yielded an observed molecular mass of 15,168.01 Da, and its N-terminal sequence was MGLTGAQIDAIK. The other amino sequence fragments exhibited a theoretical molecular mass of 15,120.67 Da, and this finding is consistent with that of amino acid sequences subjected to blast analysis. Therefore, rUuHb-F-I is the same as UuHb-F-I. With AMP prediction (CAMPR3 Collection of Anti-Microbial Peptides, http://www.camp.bicnirrh.res.in/predict_c/hii.php), many fragments in UuHb-F-I are predicted as AMPs by the Support Vector Machine classifier. For example, GLTGAQIDAIKGHWFTNIKG in positions 2–21 exhibits AMP probabilities of 1.0 (nucleotide acid sequence) and 0.873 (peptide sequence). Nevertheless, the hydrolysis of rUuHb-F-I should be further investigated.

In the current research, G^+^, G^−^, and fungus, especially common pathogenic species in aquaculture, such as* V. alginolyticus *and* V. parahaemolyticus*, may help elucidate the innate immunity of Uu. Bao et al. [12] indicated that Tg-HbI (Hb dimer) from* Tegillarca granosa* is involved in immune defense responses against microbial infection because the mRNA expression of Tg-HbI (Hb dimer) is significantly upregulated after* T. granosa* is subjected to* V. parahaemolyticus *challenge. Thus, our future work will conduct bacterial challenge to investigate the relationship between Hb and defense mechanisms of Uu.

In general, Hb and its fraction exhibit different antimicrobial activities against microorganisms through recombination or isolation [5]. Zhang et al. [11] reported that AJHb, derived from Hb-*α* in Japanese eel, exhibits a strong antibacterial activity against* Edwardsiella tarda*, with an MIC of 11.30 *μ*M of MIC. Srihongthong et al. [24] found that the Hb of alligator Hb exerts biological activity against G^+^ *Bacillus* species, such as* B. amyloliquefaciens*,* B. subtilis*, and* B. pumilus*. Belmonte et al. [25] showed that the MICs of Hb98-114 against* Cryptococcus neoformans* and* Candida tropicalis* are 1.6 and 2.1 *μ*M, respectively. Consistent with previous findings, our results revealed that rUuHb-F-I exhibits a wide range of inhibitory activities and broad antibacterial spectrum against G^+^ and G^−^ bacteria from nonaquatic and aquatic pathogenic species. Our results also showed that the inhibitory effects of rUuHb-F-I were stronger against G^+^ than against G^−^. By comparison, rUuHb-F-I did not affect* P. pastoris* GS115. The lowest MIC was 2.78–4.63 *μ*M observed in* M. luteus. *Therefore, rUuHb-F-I is an antibacterial protein or AMP precursor, which may exhibit functional diversities or selective antimicrobial activities. The results also suggested that* U. unicinctus*, similar to other aquaculture animals, may possess an innate peptide-dependent host defense system to eradicate microbes, as indicated by an MIC of 21.4–35.7 *μ*M against* V. parahaemolyticus. *Thus, our study provided a basis for the development of potent therapeutics or agents against* U. unicinctus* disease. Further studies on the digestion of rUuHb-F-I or its effects on other pathogens should be performed to produce highly active AMPs.

## 5. Conclusions 

This study is the first to report the full-length cDNA, prokaryotic expression, and antimicrobial activity of UuHb-F-I from* U. unicinctus.* The full-length cDNA sequence was 780 bp with an ORF of 429 bp encoding 142 AA. The amino acid sequence of the N-terminal chain of rUuHb-F-I was MGLTGAQIDAIK with a molecular mass of 15,168.01 Da. This protein exhibited stronger inhibitory effects against G^+^ than against G^−^. By comparison, this protein did not affect* P. pastoris* GS115. The lowest MIC observed in* M. luteus* was 2.78–4.63 *μ*M.

## Figures and Tables

**Table 1:** Primers used in this study.

Name	Sequences (5′-3′)	Purpose
Adaptor primer (Ap)	Containing the dT region designed by TaKaRa and adaptor primer part	3′-RACE cDNA
3′-RACE outer primer	TACCGTCGTTCCACTAGTGATTT	3′-RACE
3′-RACE inner primer	CGCGGATCCTCCACTAGTGATTTCACTATAGG	3′-RACE
Gene-specific primer (GSP1)	GGATATAGCGTTCTTTGACAAG	3′-RACE
Gene-specific primer (GSP2)	GCCCAGACTCTAACAGTTATCAGCTACTTGGAT	3′-RACE
SMARTer IIA oligo primers		5′-RACE cDNA
5′-RACE CDS primer A	(T)25VN	5′-RACE cDNA
10x universal primer	Long: CTAATACGACTCACTATAGGGC AAGCAGTGGTATCAACGCAGAGT	5′-RACE
A Mix (UPM)	Short: CTAATACGACTCACTATAGGGC	
5′-RACE outer primer	CATGGCTACATGCTGACAGCCTA	5′-RACE
5′-RACE inner primer	GCGGATCCACAGCCTACTGATGATCAGTCGATG	5′-RACE
Gene-specific primer (A1)	CATCATTACAGACCAGACAATACG	5′-RACE
Gene-specific primers (A2)	CGCTTCAAGAGTTGTCCGAAATGCTTCGTGGTG	5′-RACE
Primer P1	CAGGACGGAAGATATAGT	cDNA
Primer P2	GTCGTTGTGATGTAGCAG	cDNA
CDS-P1	GCGAGTCCATATG GGTCTTACTGGAGCTC	Recombinant expression
CDS-P2	TATACTCGAGCTTCATGGCGGCCACCAGG	Recombinant expression

**Table 2:** Antimicrobial activities and minimal growth inhibition concentrations (MIC) of the recombinant protein.

Microorganisms	MIC (*μ*M)
G^+^	
*Staphylococcus aureus*	7.72–12.86
*Bacillus subtilis*	>99.2
*Micrococcus luteus*	2.78–4.63
G^−^	
*Escherichia coli*	35.7–59.5
*Pseudomonas aeruginosa*	35.7–59.5
*Vibrio alginolyticus*	>99.2
*Vibrio parahaemolyticus*	21.4–35.7
Fungus	
*Pichia pastoris GS115*	>99.2
